# Quantitative Evaluation of Pentraxin-3 in Peri-Miniscrew Implant Crevicular Fluid in Patients Undergoing Orthodontic Treatment: A Prospective Study

**DOI:** 10.7759/cureus.36060

**Published:** 2023-03-13

**Authors:** Jitender Machawal, Om P Kharbanda, Ritu Duggal, Shyam S Chauhan, Vilas D Samrit

**Affiliations:** 1 Orthodontics, All India Institute of Medical Sciences, New Delhi, IND; 2 Orthodontics, Ramaiah University of Applied Sciences, Karnataka, IND; 3 Dentistry, All India Institute of Medical Sciences, New Delhi, IND; 4 Biotechnology, All India Institute of Medical Sciences, New Delhi, IND

**Keywords:** elisa, miniscrew implant, peri miniscrew implant crevicular fluid, pentraxin-3, biomarker

## Abstract

Objective: To assess the levels of Pentraxin-3 (PTX3) in peri-miniscrew implant crevicular fluid (PMICF) before and after orthodontic force application

Material and Methods: This study included 40 miniscrew implants (MSI) sites in 11 orthodontic patients with high arch discrepancy requiring first premolar extraction using maximum anchorage mechanics for the retraction of anterior teeth. After alignment, the en-masse anterior retraction was carried out using the MSI-supported direct anchorage method. PMICF was collected from the crevice of MSI using Periopaper strips 1.2µl (*Oraflow* Inc. USA) after one hour, 24 hours, and three weeks of MSI insertion and after one hour, 24 hours, seven days, three weeks, and six weeks of the force application. Samples were quantitatively analyzed for PTX3 levels through enzyme-linked immunosorbent assay (ELISA).

Results: The trend in the change of PTX3 levels was evaluated using the Wilcoxon signed-rank test. The mean concentration of PTX3 immediately after MSI insertion was 1.19 ng/ml, significantly higher than after 3 weeks after MSI insertion (0.72 ng/ml), which may correspond to the baseline. After loading, the mean PTX3 concentration increased significantly with the peak at 24 hrs (1.28 ng/ml), followed by a gradual decline till the completion of the study (0.5 ng/ml).

Conclusion: After MSI insertion, a rise in PTX3 levels in PMICF suggests an underlying inflammatory process. The slow decline in PTX3 level and return to the baseline after loading suggests an adaptive bone response to the stimulus.

## Introduction

Absolute anchorage, which is required in high discrepancy cases, can be achieved using temporary anchorage devices (TADs) [1]. Various tooth movements like mesial or distal movement, anterior retraction or protraction, intrusion or extrusion, correction of the midline, transverse problems, open bite or deep bite, skeletal anchorage for mandibular or maxillary advancement, and alternative in surgical cases can be achieved by the use of TADs [2]. Miniscrew implant (MSI), which comes under the category of TADs, has become increasingly popular because of their small size, facilitating ease of placement in basal jaw bones in multiple numbers. The most critical factor for the success of a mini-implant is primary stability [3]. The reported success rate of MSI is 70.73% to 91.6% [4]. Peri-implantitis (bone loss around the MSI and soft tissue inflammation) accounts for 30% of implant failure [5]. Clinically, it is appreciated by increased probing depth, pain, and radiographic bone loss around the MSI sites. Initially, soft tissue inflammation (reversible) occurs, which can progress to peri-implantitis if left untreated [6]. So, for the patients being treated with MSI, early identification of any tissue reaction that is harmful around the MSI is critical for the success of therapy [7].

Peri-miniscrew implant crevicular fluid (PMICF) contains various inflammatory mediators, enzymes, and tissue breakdown products, similar to gingival crevicular fluid [8]. Numerous studies have observed biomarker levels of IL-1, IL-2, IL-6, IL-8, IL-1β, OPG, and receptor activator of nuclear factor kappa beta (NFkB ligand) (RANKL), glycosaminoglycans, mainly chondroitin sulfate in PMICF following orthodontic treatment. It can be concluded that studying host response by analyzing PMICF offers a non-invasive method to detect any early indication of active disease around MSI [9-12].

One of the long pentraxins is Pentraxin-3 (PTX3), also known as the tumor necrosis factor-stimulated gene 14 (TSG14), which is synthesized in numerous cells like endothelial cells and innate immune cells infiltrating the inflammatory sites [13]. Normal PTX-3 blood level in humans is low (<2 ng/ml), but it rises quickly in inflammatory situations [14]. Recent studies indicate that the level of PTX-3 in GCF increases in response to orthodontic forces and periodontal remodeling and can be used as a possible early biomarker to examine the host reaction [15]. The present study was carried out to assess the changes in PTX-3 levels in PMICF as an inflammatory indication and prognostic marker for the stability of MSI after orthodontic force application.

## Materials and methods

The study was conducted at 40 mini screw implant sites (22 in maxilla, 18 in mandible) in 11 patients (9 females and 2 males), with a mean age of 20.27±7.21 years, cases diagnosed with high arch discrepancy requiring first premolar extraction in maxilla or mandible or both as per the treatment plan of class II div 1 or bimaxillary protrusion. The inclusion criteria used in this study were: Patients requiring fixed mechanotherapy with first premolar extractions with maximum anchorage requirement; Patients with healthy periodontal conditions; Patients with no history of systemic disease, hormonal imbalance, or drug intake (antibiotics or anti-inflammatory) during the previous 6 months; Patients with positive informed consent.

Ethical clearance was obtained from the Ethics Committee of All India Institute of Medical Sciences, New Delhi, with letter reference number IECPG/103/30.12.2015 dated 01 January 2016. Written consent was obtained from each patient before being included in the study.

**Figure 1:**
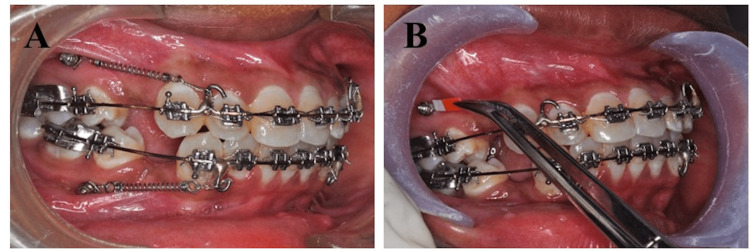
A) En-masse retraction of anterior teeth using direct anchorage through MSI, B) PMICF sample collection using Periopaper

A pre-adjusted edgewise appliance (McLaughlin-Bennet-Trevisi (MBT), 0.022" slot) was used in all the patients. After extracting the first premolar, leveling and alignment of the maxillary and mandibular arches were carried out till 0.019″ × 0.025″ stainless steel wire could be passively inserted. J-hook was soldered on the archwire (0.019″ × 0.025″) between the lateral incisor and canine area. Before MSI insertion, patients have advised betadine rinses, and the implant site was isolated with cotton rolls. Under local anesthesia, self-drilling MSIs (8 mm in length, 1.5 mm in diameter, tomas®-pin SD 08, Dentaurum Inc, USA) was positioned at 45° -60° to the long axis of the tooth in the buccal inter radicular bone in the attached gingiva between the second premolar and first molar. The 8 mm length of mini screw was chosen as the probability of success of an 8mm length mini screw is 5.7 times higher than when it is 10 mm [16]. A 9.0-mm nitinol closed coil spring delivering a 200-g force, measured using Dontrix Gauge, was used after 3 weeks of MSI insertion. The en-masse retraction of the anterior was started through the direct anchorage method (Figure 1).

**Figure 2:**
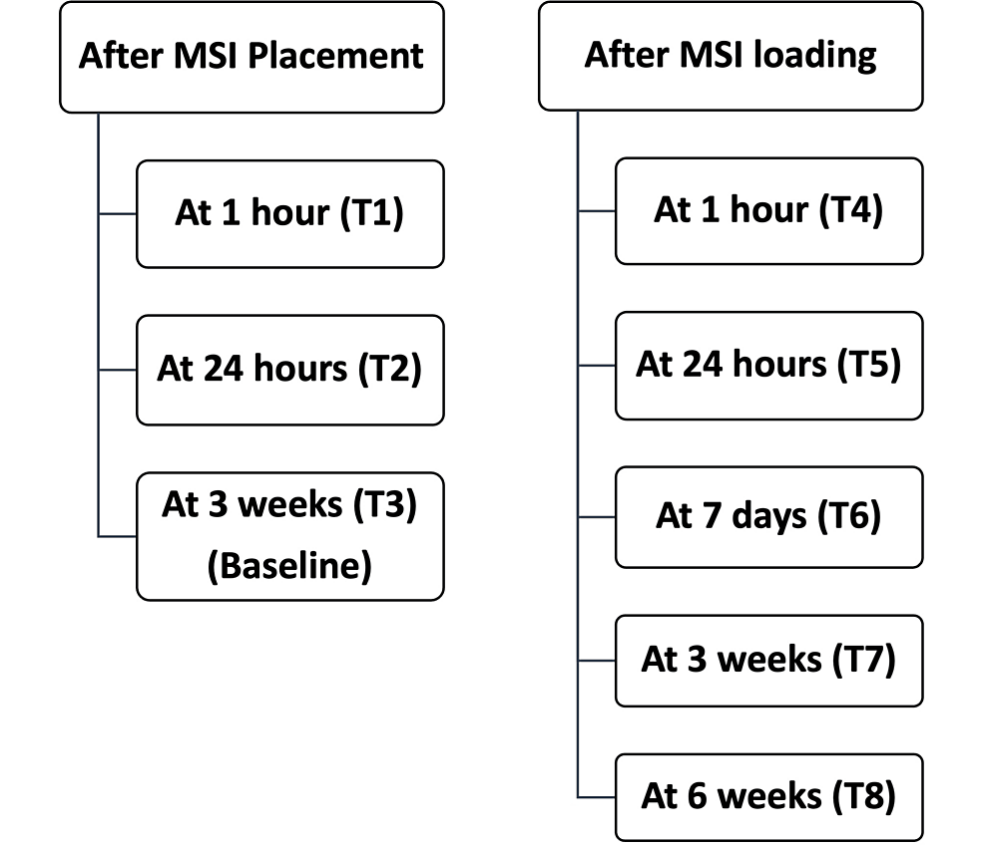
Schedule for PMICF sample collection PMICF- peri-miniscrew implant crevicular fluid; MSI- Miniscrew implants

The patients were advised to maintain good oral hygiene by brushing their teeth with a soft brush after every meal and rinsing twice daily with 0.2% chlorhexidine gluconate. They refrained from taking Non-steroidal anti-inflammatory drugs (NSAIDs) during the study. PMICF samples were collected using Periopaper strips 1.2µl (Oraflow Inc. USA) at the implant sites at scheduled time points (Figure 2).

After a vigorous gargle with sterile water, the cheek retractor was placed, and implant sites were isolated using cotton rolls. The periopaper strips were inserted into the MSI crevice until slight resistance was felt and left for 90 seconds in place. Strips that had been contaminated with saliva or blood macroscopically were discarded. The collected samples were placed in plastic sealable Eppendorf tubes and stored at -80℃ till analysis. Periotest™ (Bensheim, Germany) assessed MSI mobility during every sample collection. PTX-3 levels were estimated using RayBio (Norcross, GA, USA) Human PTX-3 Elisa kit. The standard curves for PTX-3 were drawn to quantify PTX-3, demonstrating the direct relationship between optical density and cytokine concentration.

## Results

Data were analyzed using the software Stata v22.0. First, the normality of data was analyzed using the Shapiro-Wilk test, and the data showed non-normal distribution. Therefore, Wilcoxon signed-rank test was used to assess the change in the PTX-3 levels at different observation times. A total of 3 mandibular implants failed; hence they were excluded from the study. The data obtained from 22 sites in the maxilla and 15 sites in the mandible was tabulated with a mean and median value (Table 1).

**Table 1:** PTX-3 concentration (mean and median) in Peri-Miniscrew Implant Crevicular Fluid (PMICF) between time points (T1-T8) after placement and loading of MSI. PMICF- Peri-Miniscrew Implant Crevicular Fluid; MSI- miniscrew implants

Time Points^a^	Mean (±SD)	Median (IQR)	Paired Comparison of Mean (T1-T8)^a^
T1	1.19(0.15)	1.17(0.25)	T3 vs T1 ****
T2	0.88(0.09)	0.87(0.16)	T3 vs T2 ****
T3	0.72(0.09)	0.7(0.10)	
T4	1.28(0.27)	1.32(0.51)	T3 vs T4 ****
T5	2.36(0.4)	2.31(0.63)	T3 vs T5 ****
T6	1.28(0.28)	1.25(0.40)	T3 vs T6 ****
T7	0.67(0.06)	0.68(0.05)	T3 vs T7 *
T8	0.5(0.09)	0.52(0.14)	T3 vs T8 ****
^a ^T1 indicates 1 hour after implant placement; T2 24 hours after implant placement, ; T3 3 weeks after implant placement, ; T4 1 hour after implant loading, ; T5 24 hours after implant loading, ; T6 7 days after implant loading, ; T7 3 weeks after implant loading, ; T8 6 weeks after implant loading, ; SD, standard deviation; IQR, Interquartile range ^b ^Wilcoxon signed-rank test. * P ≤ .05; ** P ≤ .01; *** P ≤ .001, **** P ≤ .0001.			

**Figure 3:**
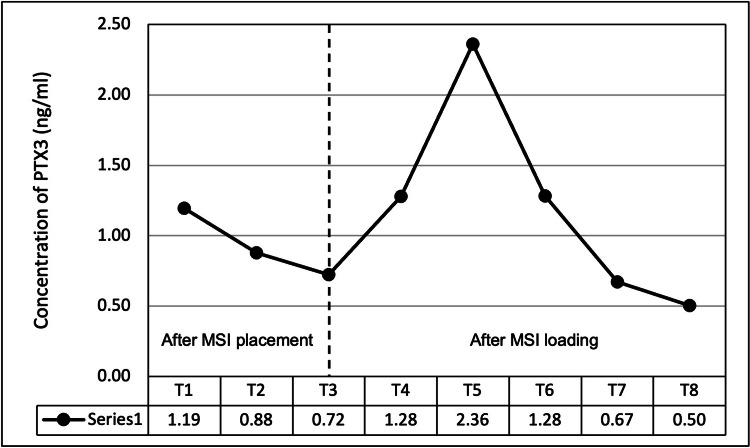
PTX-3 concentration (mean) after placement and loading of MSI PTX3- Pentraxin-3; MSI- Miniscrew implants

The PTX-3 level at T3 was considered as the baseline. The mean PTX-3 concentration was 1.19 ng/ml in PMICF 1 hour after MSI placement (T1), which gradually decreased after 24 hours (T2- 0.88 ng/ml) and 3 weeks (T3- 0.72 ng/ml) of implant placement. At 1 hour (T4) and 24 hours (T5) after implant loading, the mean concentration of PTX-3 increased to 1.28 ng/ml and 2.36 ng/ml, respectively. The mean concentration of PTX-3 after 1 week (T6) of implant loading decreased to 1.28 ng/ml. The mean concentration of PTX-3 further reduced to 0.67 ng/ml and 0.5 ng/ml after 3 weeks (T7) and 6 weeks (T8) of implant loading, respectively. The mean concentration of PTX-3 was statistically significant at all time points except T7 when compared to baseline (T3) (Figure 3).

## Discussion

Different indicative parameters, including the MSI mobility assessment, bleeding upon probing, probing depth, and gingival index, are used to assess the stability and degree of mucosal disease affecting MSI [17,18]. The clinical sign of failure comes only when the state of irreversible and incurable has occurred. So, the diagnostic parameters should be sensitive enough to detect the early changes that lead to implant failure. Furthermore, developing studies on biological markers shed light on bone remodeling and the condition of periodontal and peri-implant tissues. The levels of inflammatory mediators in the oral fluid also serve as an indicative parameter for peri-implantitis. Although there are studies describing the change in the concentration of various biomarkers in PMICF during the orthodontic force application [9-12,19,20], the present study is the first to evaluate the changes in concentration of PTX-3 in PMICF. This prospective study was conducted based on the assumption that changes in the clinical stability of MSI must be reflected in the activity of the underlying biomarkers. The age and sex of the patients were not taken into account when the samples were pooled in this investigation because studies in the literature found that neither of these variables affected the activity of enzymes [21]. 

It was statistically significant that in the present investigation, PTX-3 levels peaked at 1 hour after MSI insertion. MSI insertion causes traumatic damage to the soft and hard tissues. Following tissue damage, the organism reacts with inflammation to limit the damage or to replace the lost or damaged tissue with the processes of regeneration or reparation [22]. Interleukin-1 (IL-1) and tumor necrosis factor-alpha (TNF-a) mediated inflammatory signals strongly induce the production and release of PTX-3. Numerous cell types, including macrophages, dendritic cells, fibroblasts, activated endothelium cells, and smooth muscle cells, release PTX-3 during inflammatory activity [23]. Since MSI insertion is an invasive procedure, it elicits an inflammatory response to release the inflammatory mediators. This correlates well with the initial PTX-3 surge in the PMICF. Although miniscrew can be immediately loaded [24], we intentionally delayed it for 3 weeks so that inflammation caused by surgical trauma subsided. A significant decrease in PTX-3 levels after 3 weeks of MSI insertion indicates the sites were inflammation-free. This PTX-3 level 3 weeks after mini-implant insertion was considered the baseline level. 

The second peak of PTX-3 in PMICF was observed after 24 hours of loading of the MSI, which shows that the cells within the alveolar bone produce PTX-3 in response to the orthodontic stimulus [15]. The fact that mechanical stress may elicit biochemical and structural responses in a range of cell types, both in vivo and in vitro, can account for this rise in the PTX-3 level following the loading of MSI [25]. The PTX-3 levels then gradually decreased throughout the observation. It may be because periodontal tissues adapt to the applied orthodontic force, which also works to reduce the inflammatory mediator (feedback mechanism) [26].

In the present study, three mandibular MSIs failed and hence were excluded. A higher number of MSI failures in the mandible has also been reported in another study [27]. The larger bone density of the human mandible is thought to be responsible for the increased insertion torque, overheating of the bone, and decrease in cortical bone formation around the MSI, all of which contribute to the more failure rate in human mandibles. Failure may also be exacerbated by decreased accessibility for collecting and storing saliva due to the opening of the salivary gland ducts [28]. In the present study, we observed two peaks in PTX-3 levels: 1 hour after MSI placement and 24 hours after force application on implants for tooth movement. These findings are like a study of 1L-1β levels from miniscrew sites (PMICF) [19]. Our possibilities to compare are limited, with no other available data in the literature about the PTX-3 levels in PMICF. Future studies might help clarify the exact place for PTX-3 in this complex network of substances in the PMICF during orthodontic treatment and to see whether the expression of this biomarker correlates with force and velocity of tooth movement. This study has two major limitations: First, the study was of short duration with small sample size, and second, only a single force level was used to evaluate the effect of a mini screw implant on tissue. Therefore, a study with a longer study period with a large sample size and different amounts of force to conclude the effect of implant tissue reaction is greatly warranted.

## Conclusions

The rise in PTX-3 levels in PMICF was significant during MSI insertion and 24 hrs after loading of MSI, and this could be explained as the MSI insertion is an invasive (trauma-induced) procedure. The pattern of decreasing PTX-3 levels in PMICF during loading to the baseline shows adaptive stimulus-response from the bone. From the above results, the authors concluded that significant alteration of PTX-3 levels in the PMICF occurs during orthodontic treatment, showing the adaptive response of bone following continuous orthodontic force application. However, further studies are recommended.
